# Living environment and self assessed morbidity: a questionnaire-based survey

**DOI:** 10.1186/1471-2458-7-223

**Published:** 2007-08-30

**Authors:** Asim Saha, Pradip Kulkarni, Habibullah Saiyed

**Affiliations:** 1 Department of Occupational Medicine, National Institute of Occupational Health, Ahmedabad, India; 2 Department of Biostatistics, National Institute of Occupational Health, Ahmedabad, India; 3 National Institute of Occupational Health, Ahmedabad, India

## Abstract

**Background:**

Health complaints have been reported to be higher among the industrial area residents when compared with reference community.

**Methods:**

Such reports being only a few, a questionnaire survey was conducted in three different areas (Industrial, Residential, Commercial) of Ahmedabad city of India to determine the pattern of morbidity and to do a comparative analysis of different areas within the city.

**Results:**

A total of 679 families (243 from commercial, 199 from residential and 237 from industrial area) were included in this study. This study revealed that apart from presence of industry in close proximity to residence (99.2%), industrial area residents are having many other disadvantages from the point of view of public health like waste water stagnation (87.4%), problem of cooking smoke (33.2%) and presence of garbage dumps near residence (72.8%). Consequently, problems like coughing, wheezing, eye irritation, skin irritation, jaundice, asthma, and dental caries have been observed to be more common in industrial area. Comparative risk calculated in terms of odds ratio for different such problems have ranged from 1.83 to 6.2 when industrial area was compared with commercial area. Similarly on comparison of industrial area with residential area, odds ratio for different problems have ranged from 1.82 to 11.5.

**Conclusion:**

This study has pointed out the need of separate planning and implementation of specific upliftment programs for addressing the environmental as well as public health issues of industrial localities.

## Background

In India, urban population is exposed mainly to ambient air pollutants from automobile exhaust and industrial activities. Pollutants like SO_2_, NOX, particulate matter and volatile organic compounds like Benzene, Formaldehyde, Butadiene etc. can affect human health. Though respiratory system usually bears the main brunt of air pollutants, many other disorders involving other organ systems even cancers are attributed to air pollution. Some of the pollutants are toxic to the hematopoietic system [1]. In addition to pollution, cigarette smoke contributes significant amounts of noxious substances rendering smokers more vulnerable in comparison to the non-smokers [2]. Apart from ambient air, urban people also suffer from the problems of water pollution and the adversities of their living environment. Lack of sanitation and personal hygiene play the role of additives and contribute to the sufferings of the city dwellers. Many studies have already shown the adverse effects of pollution that have affected the health of urban people [3-5]. In urban area also, some localities are principally industrial and some others are mainly commercial or residential. The problems also differ in relation to the variations of the localities. A comparative analysis of these areas in relation to the morbidities of the dwellers can be important in assessing the real scenario of urban environmental health. In India, this kind of studies being very rare, an effort has been made through this study to evaluate the health status of urban community of an Indian city, keeping in view the possible role of pollution on human health. The objective of this study was to determine the pattern of morbidity among the city dwellers as well as to do a comparative analysis of different areas within a city.

## Methods

A questionnaire survey was conducted in three different areas (Industrial, Residential, Commercial) of Ahmedabad city. A total of 679 families (243 from commercial, 199 from residential and 237 from industrial area) were included in this study. Two-stage random sampling was done to select the study subjects. Initially three areas were selected (one area each from industrial, commercial and residential) from the city map and afterwards selection of families was done by systematic random sampling. The city of Ahmedabad is one of the prominent cities of India and is run administratively by Ahmedabad Municipal Corporation. The city is divided into three main areas by the municipal corporation for administrative purposes (residential, commercial and industrial). Industrial area is meant for industrial establishments, commercial areas for business establishments and residential area is marked for residential localities. However these demarcations are not absolutely clear-cut. Though industrial areas are studded with different industries, some families are found to reside in these areas also. Commercial areas are mainly filled with business establishments but considerable number of families does reside in those areas also. Residential areas are full with residential accommodations though some commercial establishments are also found in those areas. However, so far as ambient air pollution is concerned industrial areas of Ahmedabad city are mainly laden with industrial emissions and in commercial and residential areas, vehicular smoke is the main offender. A questionnaire was used to collect information regarding age, sex, habits, occupation, living environment, hygiene status and morbidity. This interviewer-administered questionnaire was filled up by medical personals only. Though no detailed medical examination of the subjects was done preliminary examination/assessment of the study subjects was done to facilitate diagnosis of evident easily diagnosable health morbidities. While designing the questionnaire, due emphasis was given to make the questions easily understandable, comprehensive and non-ambiguous. Questions were designed in such a way that answers to every question was very specific (mostly yes/no) and was not based on any subjective feeling/understanding of the participants. Few questions were repeated in the questionnaire in different pages, to assess the consistency of the answers given by participants. After designing the questionnaire, it was applied at first on a small group on a pilot basis by different interviews to test how the questionnaire works and then only it was used in the actual study. Data entry and analysis was done by using Epi Info 6 and SPSS 6.1.4 software. Different proportions were compared by applying chi square test. While comparing different conditions reported by the residents of different areas, odds ratios and 95% confidence intervals were calculated to examine the significance of the observed differences. Logistic regression technique was used to obtain the contribution of location of residence on the health outcome of the residents irrespective of the effect of different other possible confounding factors. Location of residence (industrial versus commercial or industrial versus residential), education level (illiterate, below university level educated and university level educated), occupation (dusty, non-dusty) and smoking status (ever smoker, never smoker) were taken as categorical variables whereas age (yrs) and per capita income (Indian rupees) were taken as continuous variables. We accommodated location of residence (either industrial versus commercial or industrial versus residential) together with other possible confounding variables in the logistic regression model simultaneously in order to estimate the effect of location of residence, adjusting for the effects of other variables. Necessary ethical clearance was obtained from the institutional ethics committee of National Institute of Occupational Health, India for the purpose of this study. Informed consent was obtained from each of the subjects and then only the subjects were registered for participation in this study.

## Results

Thirty six percent of families were from commercial area; residential and industrial areas contributed 29.3 and 34.9 percent respectively. Fairly good participation was observed from the study participants. Participation rate was 97% in commercial area, 91% in residential area and 96% in case of industrial area. Average number of family members per family was 4.9 in case of commercial area and 4.5 in case of residential and industrial areas (Table 1). Everywhere most of the subjects were from 20 to 39 years age group. This age group represented 40% subjects in commercial area and 36% and 37.4% subjects in residential and industrial areas respectively. Mean age of the female population under study was 29.4 years, 33.9 years and 29.2 years in industrial, commercial and residential areas respectively. The values were 28.1 years, 30.5 years and 29.2 years in case of male population. Among industrial area population, 14.4% subjects were smokers and 1.5% subjects were regular consumers of alcohol. In commercial area the number was 11.5% and 2.8% and in residential population smokers and regular consumers of alcohol were 11.7% and 1.6% respectively. Mean income of the subjects was highest in residential area followed by commercial and industrial areas. In all the areas male subjects were having more income than the females. Majority of our subjects were housewives, students and office workers. In commercial areas the percentage was 29.4, 28.9 and 23.4 respectively. In residential and industrial areas the same percentages were 24.0, 35.2, 29.8 and 22.2, 28.5, 32.6 respectively.

**Table 1:** Demographic characteristics of the study participants

**Demographic characteristics**		**Commercial area**	**Residential area**	**Industrial area**
Number of the families		243 (35.8%)	199 (29.3%)	237 (34.9%)
Mean number of family members		4.9 ± 2.2	4.5 ± 1.4	4.5 ± 1.7
Mean age	Female	33.9 ± 19.8	29.2 ± 16.6	29.4 ± 17.2
	Male	30.5 ± 18.6	29.2 ± 17.4	28.1 ± 17.8
Smokers		126 (11.5)	96 (11.7)	139 (14.4)
Regular consumers of alcohol		23 (2.1)	13 (1.6)	14 (1.5)

Around 95% of study subjects of all localities were drinking treated water. Garbage disposal facility was enjoyed by 71.3% inhabitants of industrial area whereas 97.6% and 92.1% inhabitants of commercial and residential areas respectively had the same facility. Wastewater stagnation was a problem of industrial area (87.4% subjects were suffering) only. Problem of vector was experienced more in industrial area (98.9%) in comparison to commercial (80.6) and residential areas (82.9). Seventy three percent subjects of industrial area had the problem of presence of garbage dumps near residence while the same problem was reported by 36.6% and 63.8% participants from commercial and residential areas respectively. Two other problems mostly experienced by industrial area people was presence of industry in close vicinity of residence (99.2) and rainwater stagnation (90.7). Eighteen percent of subjects of residential areas were living in slums, 45.2% in low-income settlements and 31.4% in high-income settlements. In industrial areas 90.3% subjects were living in low-income settlements. Almost 80% subjects of commercial areas were residing in low-income settlements. Main source of drinking water in industrial area was own/bore well (80.1%) and in commercial area it was municipal tap (97.6). In residential area 60% subjects were using own/bore well and 40% were using municipal tap. In all the areas majority of the houses were pucca followed by semi-pucca and kachha houses (Table 2). On comparison of hygiene status and living environment of different localities it was observed that industrial area was statistically significantly inferior to residential and commercial areas.

**Table 2:** Comparison of hygiene status and living environment of the study subjects living in different localities

**Parameters**		**Commercial area No (%)**	"Industrial versus commercial area" Significance* (P value)	**Industrial area No (%)**	"Industrial versus residential area" Significance* (P value)	**Residential area No (%)**
Source of drinking water	Municipal tap	1172 (97.6)	< 0.00001	208 (19.5)	< 0.00001	424 (40.1)
	Own/bore well	24 (2.0)	< 0.00001	856 (80.1)	< 0.00001	630 (59.6)
Treatment of drinking water		1157 (97.5)	< 0.01	1014 (95.2)	NS	855 (96.0)
Garbage disposal facility		1158 (97.6)	< 0.00001	750 (71.3)	< 0.00001	816 (92.1)
Waste water stagnation		54 (4.6)	< 0.00001	915 (87.4)	< 0.00001	39 (4.4)
Problem of vector		954 (80.6)	< 0.00001	1040 (98.9)	< 0.00001	737 (82.9)
Problem of cooking smoke		204 (17.2)	< 0.00001	355 (33.2)	< 0.00001	130 (14.4)
Presence of garbage dumps near residence		435 (36.6)	< 0.00001	763 (72.8)	< 0.0001	585 (63.8)
Industry Close to residence		19 (1.6)	< 0.00001	1054 (99.2)	< 0.00001	11 (1.2)
Rain water stagnation		64 (5.4)	< 0.00001	938 (90.7)	< 0.00001	104 (11.5)
Kachha/Semi-pucca house		424 (35.5)	< 0.00001	156 (14.6)	< 0.00001	236 (26.4)

* Significance tested by chi square test

On multivariate analysis using logistic regression model, it was found that problems like cough in morning (OR 1.83), wheezing (OR 4.2), frequent loose stools (OR 6.2), eye irritation (OR 5.8), jaundice (OR 3.1), asthma (OR 2.9) and dental caries (OR 2.1) were significantly higher in industrial population when compared with commercial area. Comparative risk calculated in terms of odds ratio for different problems have ranged from 1.83 to 6.2. Similarly on comparison of industrial area with residential area, it was found that frequent loss of appetite (OR 3.2), frequent loose stool (OR 2.6), jaundice (OR 11.5), skin irritation (OR 3.7), eye irritation (OR 3.1) and dental caries (OR 1.82) were significantly higher among industrial area population. Odds ratio for different problems have ranged from 1.82 to 11.5 (Table 3). So far as the role of different other factors on different health complaints is concerned, it was observed that apart from location of residence other factors like smoking habit and occupation had significant effect on some of the health complaints. Smokers had increased risk of cough in the morning (OR 1.64, 95% CI 1.02–2.39 in case of "industrial versus commercial area" comparison and OR 2.32, 95% CI 1.47–4.02 in case of "industrial versus residential area" comparison) and dusty occupation showed increased risk of asthma (OR 3.9, 95% CI 1.4–7.4 in case of "industrial versus commercial area" comparison).

**Table 3:** Comparison of morbidity status of the study subjects living in different localities

**Morbidity**	**Industrial area No (%)**	**Commercial area No (%)**	**"Industrial versus commercial area" Odds ratio (unadjusted) (95% CI)**	"Industrial versus commercial area" Odds ratio* (95% CI)	**Residential area No (%)**	**"Industrial versus residential area" Odds ratio (unadjusted) (95% CI)**	"Industrial versus residential area" Odds ratio* (95% CI)
Cough in morning	35 (3.7)	16 (1.6)	2.36 (1.26–4.49)	1.83 (1.05–3.29)	15 (2.0)	1.88 (0.99–3.64)	1.36 (0.79–3.21)
Phlegm in morning	22 (2.3)	10 (0.9)	2.59 (1.16–5.89)	2.12 (1.02–4.92)	11(1.3)	1.79 (0.82–3.95)	1.76 (0.72–3.47)
Breathlessness while walking	53 (5.5)	50 (4.6)	1.21(0.80–1.83)	1.32(0.72–1.94)	27 (3.3)	1.7 (1.04–2.81)	1.8 (0.94–3.87)
Wheezing	36 (3.7)	9 (0.8)	4.8 (2.2–10.7)	4.2 (1.6–9.6)	19 (2.3)	1.63 (0.90–2.98)	1.44 (0.82–3.08)
Hemoptysis	6 (0.6)	4 (0.4)	1.50 (0.38–6.34)	1.67 (0.49–5.94)	4 (0.5)	1.35 (0.34–5.70)	1.32 (0.29–4.69)
Asthma	23 (2.4)	8 (0.7)	3.5 (1.5–8.5)	2.9 (1.05–7.02)	14 (1.8)	1.34 (0.66–2.76)	1.89 (0.86–2.92)
T.B	6 (0.6)	5 (0.5)	1.20 (0.32–4.54)	1.39 (0.29–3.57)	2 (0.2)	3.01(0.55–21.59)	2.91(0.42–11.20)
Frequent loss appetite/nausea	56 (5.8)	11 (1.0)	6.1(3.1–12.4)	4.2 (2.02–8.3)	11 (1.4)	4.3 (2.2–8.8)	3.2 (1.2–5.7)
Frequent loose stools	40 (4.2)	4 (0.4)	10.9 (3.7–36.1)	6.2 (2.7–16.9)	14 (1.7)	2.5 (1.3–4.9)	2.6 (1.4–4.1)
Jaundice	16 (1.7)	6 (0.5)	3.4 (1.3–9.9)	3.1 (1.7–7.2)	1 (0.1)	17.3 (2.4–350.3)	11.5 (2.3–37.9)
Malaria	22 (2.3)	40 (3.6)	0.63 (0.36–1.10)	1.01 (0.42–1.43)	28 (3.4)	0.67 (0.37–1.22)	0.97 (0.62–1.49)
Skin – itching/redness	65 (6.7)	30 (2.7)	2.6 (1.6–4.1)	1.8 (0.92–5.3)	10 (1.2)	5.9 (2.9–12.3)	3.7 (1.2–9.3)
Eye irritation	234 (24.3)	32 (3.5)	8.8 (5.9–13.2)	5.8 (2.3–9.1)	49 (6.7)	4.5 (3.2–6.3)	3.1 (2.1–7.3)
Motting/fluorosis	22 (2.3)	2 (0.2)	11.7 (2.7–72.4)	3.9 (1.5–11.4)	8 (1.0)	2.3 (0.98–5.72)	1.9 (0.72–4.22)
Dental carries	331(34.4)	189 (17.3)	2.5 (2.03–3.09)	2.1 (1.76–3.29)	197 (24.1)	1.65 (1.33–2.04)	1.82 (1.03–2.94)

*Adjusted for age, smoking, educational status, income and occupation.

## Discussion

Industrial areas have been associated not only with industrial pollutants but also with lack of sanitation and environmental hygiene. Consequently these areas have mostly carried a significant load of public health problems. This study also, revealed that apart from presence of industry in close proximity to residence, industrial area is having many other disadvantages from the point of view of public health like consumption of water from own/bore well, waste water stagnation, problem of cooking smoke and presence of garbage dumps near residence. Consequently, problems like coughing, wheezing, eye irritation, skin irritation, jaundice, asthma and dental caries have been observed to be more common in industrial area.

Studies have already established that industrial emissions are the primary contributors to air pollution. High levels of ozone and other pollutants can cause respiratory symptoms especially asthma and chronic airway disease in susceptible individuals [6]. A relationship has been observed between somatic diseases, functional changes, dental mortality in children and their stay in urban industrial areas, where ecological condition has also been observed to be poor [7]. A study, similar in nature as the present one found that all categories of self-reported health effects were elevated, with an odds ratio > 3.0, in the inhabitants of industrial area residents when compared with the reference community [8]. Likewise in case of this present study also industrial emissions, which contain a range of harmful agents like oxides of carbon-sulphur-nitrogen, suspended particulates, volatile organic compounds etc. may possibly have major contributions to the morbidities of the industrial area residents. Industrial effluents and lack of sanitary and public health attention may also have been responsible for the inferior status of hygiene and living environment in industrial area as observed in this study. However no comparative assessment between commercial and residential area was undertaken because the distinction of this two areas was not very clear cut though the difference of industrial with other areas was very distinctive.

This study however has some limitations also. The study being based on self-reports also had potential reporting biases. The cross sectional design also prevents understanding of the temporal relation of adverse living environmental conditions and health effects. Furthermore, inclusion of greater sample size and selection of subjects from more cities could have made the findings of this study more generalisable.

This study like some other studies [9-14] has pointed out the need of separate planning and implementation of specific upliftment programs for addressing the environmental as well as public health issues of such industrial localities.

## Conclusion

Separate and specific measures are necessary to address the environmental and public health issues of industrial localities.

## Competing interests

The author(s) declare that they have no competing interests.

## Authors' contributions

***AS: ***Planned and designed the study, executed the study and prepared the final write up.

***PKK: ***Designed the study and analyzed the data.

***HNS: ***Designed the study and contributed in the write up.

All authors have read and approved the final manuscript.

## Pre-publication history

The pre-publication history for this paper can be accessed here:


